# Leveraging blue spaces for public health: Co-creating a whole-system action plan

**DOI:** 10.1016/j.puhip.2025.100665

**Published:** 2025-10-04

**Authors:** Niamh Smith, Michail Georgiou, Deryck Irving, Sebastien Chastin

**Affiliations:** a School of Health and Life Sciences, Glasgow Caledonian University, Glasgow, G4 0BA, UK; b Urban Big Data Centre, University of Glasgow, Glasgow, G12 8QQ, UK; c Hydro Nation Chair Research & Innovation Programme, University of Stirling, Stirling, FK9 4LA, UK; d Department of Movement and Sports, Ghent University, Watersportlaan 2, 9000, Ghent, Belgium

**Keywords:** Blue space, Systems mapping, Public health, Co-benefits, Policy formulation, Co-creation

## Abstract

**Objectives:**

• To co-create evidence-based, whole-system recommendations for leveraging blue spaces to enhance public health.
• To highlight the salutogenic and equigenic benefits of blue spaces.
• To guide decision-makers and practitioners in preserving, revitalising and using urban blue spaces for synergistic benefits to people and the planet.

**Study design:**

Systems based co-creation.

**Methods:**

1) Establish an evidence base through extensive mixed-methods academic research over six years on the health benefits of urban blue spaces.
2) Partner with a water-focused national research and innovation program to incorporate policy expertise and reduce siloed working.
3) Co-create an action plan with stakeholders using system-based participatory research, following the DISCOVER framework.

**Results:**

The full system map consists of 137 variables and 220 causal linkages. The system map is structured around four core mechanisms that illustrate how urban blue spaces influence health: promoting physical activity, fostering social interaction, supporting a healthy environment, and reducing population stress.

**Conclusions:**

Four strategic objectives identified, achievable through 12 policy actions. Blue spaces offer significant health benefits that have historically been overlooked in regeneration projects. The co-created recommendations provide a comprehensive guide for decision-makers and practitioners to maximise the health benefits of blue spaces. Implementing these recommendations can lead to synergistic benefits for both people and the planet.

## 1 Introduction

Globally, the prevalence of non-communicable diseases (NCDs), obesity, physical inactivity, and poor mental health is increasing. The COVID-19 pandemic has exacerbated the situation but also increased public understanding of the importance of outdoor spaces for public health [1]. Moreover, awareness of the connection between human health and the planetary ecosystem has grown as we have an increased understanding of climate change [2]. Climate change is amplifying the frequency and severity of extreme weather events, resulting in substantial disruptions to the natural and social systems that are vital for maintaining human health. Additionally, this phenomenon increases the economic, social and environmental pressures faced by individuals and communities [3].

Scotland's health and health inequality records are among the poorest in Europe, earning it the moniker the "sick man" of Europe [4]. In Scotland, physical inactivity and high obesity rates [5] contribute to the high prevalence of non-communicable diseases [5]. Furthermore, mental health concerns in Scotland are high and show disparities between deprived areas and more affluent areas [6], with the COVID-19 pandemic exacerbating mental health issues among specific vulnerable groups [7]. Scotland is also feeling the effects of climate change, particularly concerning water [8]. Scotland's national ambition to become a "Hydro Nation" [9] positions it as an ideal setting to explore the potential of blue spaces as a public health asset.

Urban blue spaces, such as rivers, canals, and coastal environments, are increasingly recognised as important components of urban planning and design, contributing to public health, ecological resilience and public health [10,11]. Maximising the health benefits of blue spaces for local populations can thus be considered a significant public health opportunity.

While existing research has demonstrated the benefits of blue spaces on urban living through empirical studies and global case study examples, questions remain about how best to manage, regenerate and use these spaces to improve health outcomes [11]. Addressing these challenges require innovative systems approaches that consider the complexity of urban environments and the diverse stakeholders involved. A systems approach is a way of understanding and addressing complex problems by examining how different components—such as people, processes, environments, and policies—interact within a larger, dynamic system, recognising feedback loops, interdependencies, and the potential for unintended consequences [12]. Such approaches have gained traction in the last decade in environmental science and health and social care [[13], [14], [15]].

Despite ongoing efforts to revitalise blue spaces, many initiatives remain ‘piecemeal’, often lacking coordination, long-term vision, and inclusive stakeholder engagement. Stakeholders range from users of blue spaces, the water industry, conservation organisations, urban planners, tourist boards, transport officials, community organisations, local businesses, public sector bodies and academics, to name a few. Such stakeholders often operate in silos, with conflicting agendas. Without a systems approach, interventions risk being fragmented, unsustainable, and misaligned with community needs [16].

Contributing to this growing field of blue space and public health research, this paper presents the evidence-based co-creation of pragmatic, informed by six years of multi-method pluridisciplinary research, and culminating in the use of the DISCOVER framework to guide the co-creation process [10,[17], [18], [19], [20], [21], [22]]. While existing models and frameworks provide valuable structures for understanding environmental determinants of health [23,24], they can be critiqued for their potential to simplify causal chains into single linear pathways and their model of consultation rather that co-creation. Co-creation in public health is increasingly regarded as a valuable approach to fostering innovation and enhancing stakeholder engagement [25,26]. By actively involving relevant parties in the development process, co-creation has been shown to facilitate the implementation and uptake of evidence-based interventions [27]. The DISCOVER framework embeds systems-based co-creation methods as a valuable means to integrate different perspectives and visualise their interconnectedness [28]. These recommendations can support decision-makers to preserve, revitalise and use urban blue spaces in a way that provides synergistic benefits to people and the planet.

## 2 Methods

### 2.1 Context

**Fig. 1:**
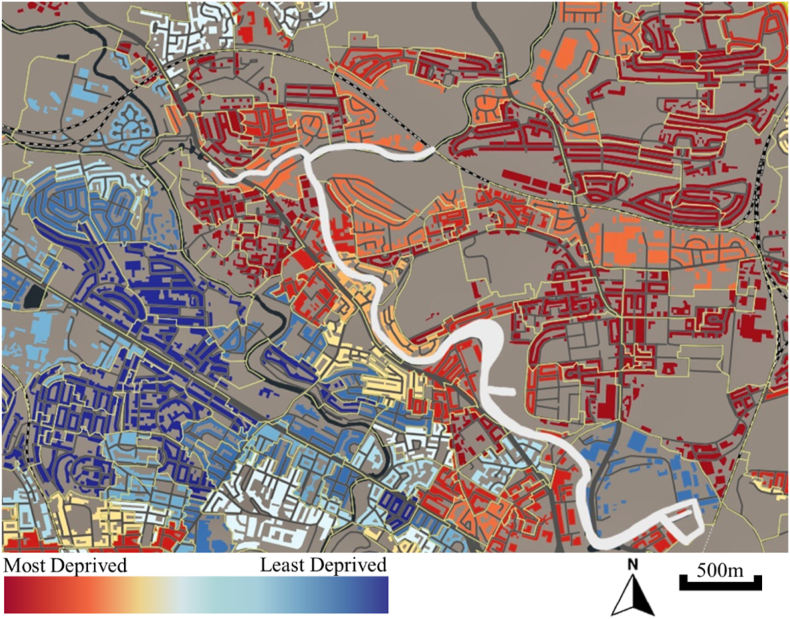
Map of the canals in North Glasgow showing Scottish Index of Multiple Deprivation (SIMD).

Since the early 2000s, the canals in North Glasgow have undergone significant regeneration, including the Smart Canal project to address flooding, new housing developments, and the creation of recreational and cultural amenities such as a watersports complex, nature reserve, skatepark, and creative hub. The construction of the Stockingfield Bridge has further improved connectivity between three communities and the city centre. Despite these developments, North Glasgow remains one of the most socioeconomically deprived areas in the city (Fig. 1), with higher levels of poverty, unemployment, and poor health outcomes compared to more affluent neighbourhoods [29] and also experience lower-quality greenspace than wealthier neighbourhoods [30]. Health was not a primary objective of the regeneration efforts; however, longitudinal research has shown that living within 700 m of a blue space in deprived areas is associated with a 3 % reduction in annual mortality, a 10–12 % lower risk of obesity and diabetes, and a 15 % reduction in cardiovascular-related conditions [20]. A follow up study found that proximity to blue space can buffer the mental health impacts of socio-economic deprivation [18], and qualitative research highlights the therapeutic value of the canals as community assets [18,21].

This regeneration context provided a unique natural experiment, as the canal improvements were the only major intervention in the area. Given the compounded health and socio-economic challenges in North Glasgow [23,24], exposure to natural environments like blue spaces may help alleviate health pressures and moderate the effects of deprivation [24].

### 2.2 Study Design

We followed a three-step process to arrive at our recommendations: establishing an evidence base, forming a partnership, conducting systems-based co-creation.

1) Established an Evidence Base

Before initiating the co-creation process, we conducted extensive mixed-methods research to explore the health benefits of urban blue spaces, combining evidence synthesis and evaluation of the regeneration of the canals in North Glasgow as a natural experiment, from 2016 to 2021. We conducted desk reviews to quantify the impact of blue spaces on health, the mechanisms linking blue space and health and existing 'blue prescription' initiatives [10,17,22]; citizen science employing the Our Voice tool to understand local peoples' needs [31]; qualitative research on canal usage and features that promote blue spaces as therapeutic landscapes [18,21]; longitudinal epidemiological data analyses to quantify the impact of regenerating blue spaces using 18 years' worth of routinely collected NHS data on physical and mental health outcomes [19,32]; and a valuation of the blue space benefits of mental health to assess their potential cost-effectiveness as therapeutic sites for the treatment of mental health conditions.

2) Formed a partnership with Hydro Nation

As the Scottish Government's initiative to maximise the value of the country's water resources, Hydro Nation offered a unique opportunity to embed our work within a broader policy framework focused on sustainability, innovation, and public benefit. Its established networks bring together a wide range of stakeholders, which provided beneficial connections to involve co-creators. This partnership allowed us to improve the communication and dissemination of our research, helping us reach a wider audience with our policy recommendations.

3) System-based co-creation using the DISCOVER framework

**Fig. 2:**
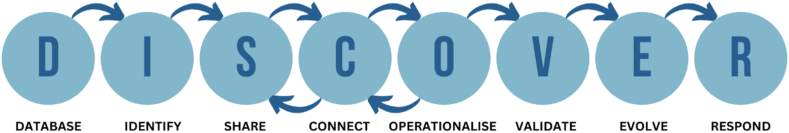
DISCOVER framework for planning, implementing and governing systems-based co-creation. Arrows show direction of travel.

We applied DISCOVER as a strategic, eight-step framework that provides an actionable, systematic way to address complex problems using participatory cocreation. The DISCOVER process began in 2021, with the final co-creation session taking place in October 2022. The eight steps of DISCOVER are followed sequentially but can overlap, allowing for iteration as understanding evolves (Fig. 2Figure 2). A previous paper details the development of DISCOVER [28]; this paper focused on presenting the findings and recommendations. Throughout this methods section, the steps of DISCOVER are italicised for clarity.

**Fig. 3:**
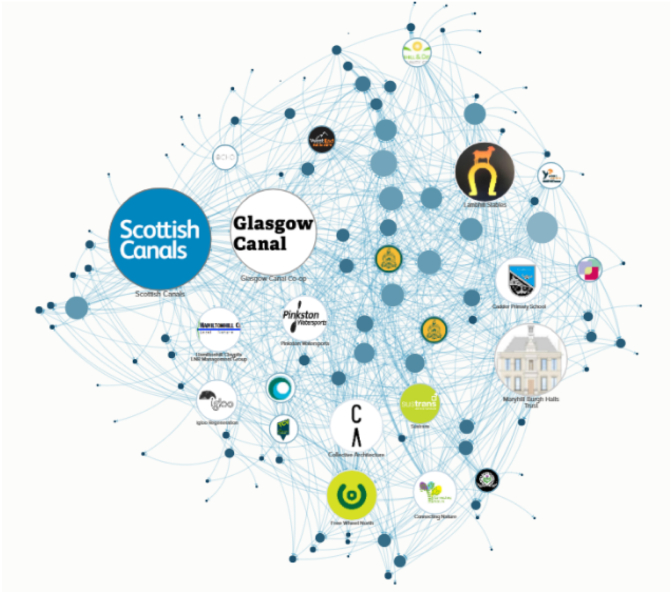
Network map of stakeholders operating around the canals in North Glasgow.

The first stage of DISCOVER was to establish an exhaustive stakeholder map of people and organisations operating around the canals in North Glasgow. We compiled a comprehensive *database* by conducting a systematic search and using university contacts, Google maps, and partner organisations. Sociometric data was gathered through an online survey, including collaboration ratings and interest in working together in the future. The survey was distributed through email, social media, and follow-up emails. Data was visualised as a network using KUMU (Fig. 3). This process enhanced our familiarity with different organisations and informed our co-creation sessions.

We then devised sampling criteria to support *identifying* co-creators to invite to co-creation sessions. Guidelines recommend involving 10–12 co-creators to account for potential dropouts [33]. We engaged eight researchers in an initial workshop, before meeting with individuals and groups to discuss the factors that contribute to blue spaces as being health-enhancing or health-limiting. The researchers worked through the *share* and *connect* steps using traditional Group Model Building (GMB) approaches to develop causal loop diagrams (CLDs) [34,35]. GMB is a collaborative approach used to engage stakeholders in mapping and understanding complex systems [35]. Co-creators *shared* all key variables that should be considered when thinking about blue spaces and health based on evidence garnered from research, their lived experiences or professional expertise. They then discussed the *connections* between the different variables and drew lines between these to form a complex map.

We then presented this model to individuals and groups of stakeholders via individual online meetings to discuss what was missing in order to *operationalise* the map. This approach was most appropriate due to scheduling constraints during the COVID-19 pandemic. A list of the co-creation methods, the range of co-creators involved in each step and an example of the key questions asked in each session is shown in Table 1. To ensure representation of local perspectives, we built relationships with a community organisation and engaged individuals with deep, lived experience of the area. These included a long-standing community volunteer, a local primary school teacher familiar with youth engagement along the canals, a resident active in local nature reserve associations, and a respected manager of a community-led organisation. Their insights directly shaped the systems map, which was then *operationalised* and *validated* through broader stakeholder engagement in the final co-creation workshop.

**Table 1:** List of co-creators involved in the system map.

Co-creation Methods	Co-creators Involved	Main prompts and screenshots of Co-creation Output
Initial Group Model Building Process (*share* and *connect*) to build a systems map based on the existing literature and research expertise.	9 x researchers with specialties in social work, physical activity, blue space, engineering, occupational therapy, physiotherapy.	What are all of the factors that link Blue Space and Health?
Individual/small group co-creation sessions conducted online due to Covid-19 restrictions limiting face-to-face contact and difficulties scheduling online group meetings. Systems maps presented and narratively explained. Discussed missing factors. The maps were *operationalised* after each interaction. Member checking continuously (*validate*) and final map shared with all co-creators.	Local resident and regular user of the Canals	What is missing?
	Local primary school teacher	
	1) Community representatives from a local nature reserve 2) Architect who had worked on waterfront redevelopments	
	1) Employee at a local community hub 2) Volunteer at a local community hub	
	Manager at local community-led organisation	
	Director of an urban design organisation	
	Representative of a public body responsible for Scottish waterways	
Co-creation of the recommendations in an online co-creation workshop. Final *evolved* map presented. Recommendations brainstormed and categorised. Output synthesised by the research team into a set of 12 recommendations (*respond*).	Representatives from - two local housing associations - a public corporation that manages land - a third sector working in health and social care - other universities -a sustainable transport charity -two national water agency -national environmental protection agency -government agencies -city council	1) What existing efforts have been made to improve health using blue spaces? 2) What other actions could be taken? 3) Plot these on an Action Priority Matrix – Quick Wins and Major Projects. 4) Breakout rooms to further discuss. 5) Research team synthesised the output from these sessions to finalise the recommendations. These were member checked.

To *validate* our findings and ensure trustworthiness, we used triangulation, using multiple data sources, co-creation session outputs, and follow-up emails and interviews to member-check our findings [36]. By making the entire system map creation process participatory and iterative, we ensured a robust and reliable representation of the relationships between blue space and health.

The map *evolved* following the development, refinement, and analysis of the system map for the blue space and health system, which enabled the identification of key pathways and leverage points for potential interventions to influence the system's functioning. The data generated from DISCOVER aligned with the four key mechanisms linking blue space and health: physical activity, social interaction, stress reduction, and environmental conditions. Actions addressing these mechanisms were likely to bring about positive change as they aligned with existing evidence and co-creators' worldviews.

The final *respond* step was actioned through a final 2h online co-creation workshop. Key decision-makers working around blue spaces and health were invited, via email, to participate. We had a total of 22 attendees at our final online meeting (Table 1).

The co-creation workshop aimed "To co-create a Blueprint for Improving Health and Health Inequalities using Blue Spaces". Following this final session, the proposed ideas were analysed thematically by the research team and grouped into actionable recommendations [37]. The *respond* step had two main outputs: this academic paper and the policy recommendations titled "Leveraging Blue Spaces for Health: A blueprint for action", launched at a national event organised for World Water Day 2023.

Ethical approval was obtained from the School of Health and Life Sciences at Glasgow Caledonian University (HLS/PSWAHS/19/208).

## 3 Results

**Fig. 4:**
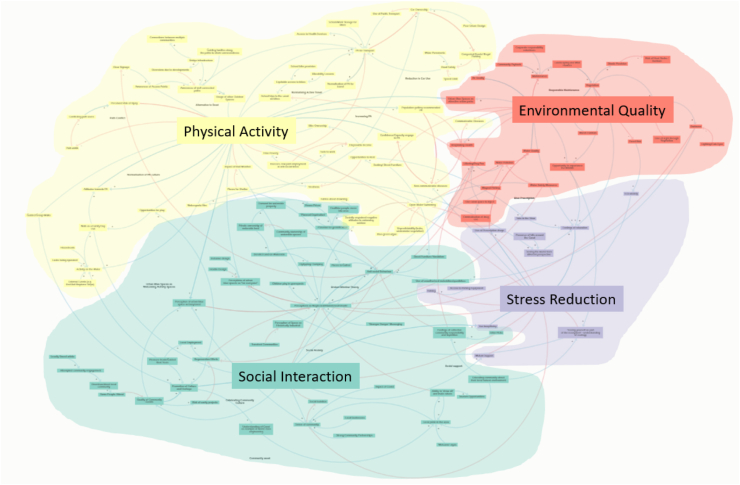
System map of the factors which contribute to blue space relationship with population health.

The full system map consists of 137 variables and 220 causal linkages. The system map is structured around four core mechanisms that illustrate how urban blue spaces influence health: promoting physical activity, fostering social interaction, supporting a healthy environment, and reducing population stress. These mechanisms form the central framework of the map, which is based on findings from interviews and co-creation sessions with stakeholders involved with canals and waterways in Scotland. Surrounding these core elements is a network of interconnected variables that either directly or indirectly impact public health outcomes. To understand the map, it is helpful to break it down by these mechanisms, each of which includes key factors, relationships, and feedback loops. Fig. 4 presents the full map, color-coded by mechanism, and an interactive version is available online (https://nsmith222.kumu.io/recommendations-for-leveraging-blue-space-for-health). A detailed narrative summary is provided in Supplementary Information 1.

## 4 Discussion

**Fig. 5:**
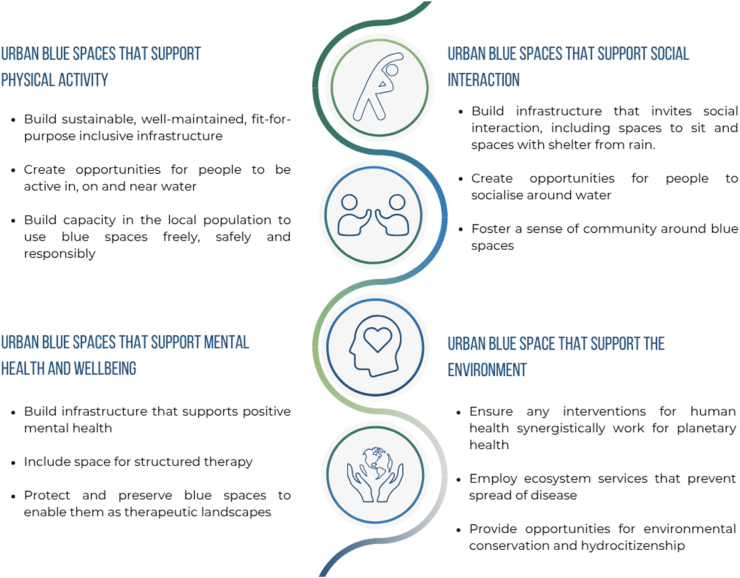
Infographic highlighting the 12 key actions in which blue space can play a role in promoting public health.

These recommendations are the first actionable recommendations for leveraging blue space for public health, including the experiences and expertise of around 300 people and longitudinal evidence covering 19 years of data. The recommendations policy document is available here: https://zenodo.org/record/7756047#.ZBnK8nbP1Pb. An infographic detailing the recommendations is shown in Fig. 5.

### 4.1 Physical activity

The first objective relates to space for physical activity. Studies have shown that being near or interacting with blue spaces can lead to increased physical activity, which in turn can improve cardiovascular health and reduce the risk of chronic diseases such as obesity and diabetes [10,17].

We propose three policy actions to promote physical activity around blue spaces.

The first action is to build sustainable, well-maintained, and inclusive infrastructure that is suitable for people of all ages and abilities. This may include wide, well-maintained paths, designated cycling paths, and clear signage to emphasise the connectedness of paths. Wide, well-maintained paths that are accessible for people with mobility challenges and people with prams may increase usage [21]. Designated cycling paths may be added to ensure the safety of both cyclists and pedestrians. Finally, clear signage that emphasises the connectedness of paths can help people navigate the area easily and reduce confusion.

The second action is to create opportunities for people to be active in, on, and near water, such as open-water swimming and equitable opportunities for watersports like paddleboarding, canoeing, and kayaking. These activities are not only enjoyable but also good forms of physical activity while also providing a sense of adventure and excitement. Increasingly there are charities, social enterprises and community organisations working to improve access to water-based activities, for example, The Sorted Project, which supports people in recovery on a canal boat in Edinburgh and Bridge 8 Hub, a canal-based outdoor activity hub on the Union Canal in Edinburgh. Open-water swimming is another opportunity that can be considered, which can be enjoyed by people of all ages and abilities when done in safe environments. Research has indicated that wild swimming promotes mindfulness, builds resilience and allows people to become more attuned to their bodies [38] while also having a social element, and is thus connected with the stress reduction and socialisation areas of the system map.

The third action is to build capacity in the local population to use blue spaces freely, safely, and responsibly, for example, by providing bike-ability lessons, bike storage facilities, and safe spaces for free play. Cycling lessons may contribute to increased confidence and capacity for people to use the paths along blue spaces to cycle for active travel and leisure. In Scotland, all children undertake 'Bikeability Training' designed to provide the skills and confidence they need to cycle safely on roads [39]. Providing suitable bike storage facilities can make it easier for people to cycle to blue spaces and park their bikes safely [40]. Additionally, creating safe spaces for free play, such as playgrounds or outdoor gyms, can help encourage people to visit the blue space and engage in physical activity. In contrast, if infrastructure improvements are made without engaging with community members, or equitably establishing opportunities and capacity for locals to use the spaces, there is a potential risk of exacerbating health inequalities.

These policy actions and infrastructure changes have the potential to improve physical health outcomes and increase access to blue spaces for all individuals.

### 4.2 Social interaction

Blue spaces can provide opportunities for social interaction and community building, which in turn can have a positive impact on mental and physical health [18,41,42]. Even simply walking or sitting by the water can provide opportunities for social interaction with friends or strangers. We propose three policy actions to position urban blue spaces as social spaces.

First, build infrastructure that invites social interaction, such as seating and shelters. Effective seating encourage psychological comfort, physical comfort and pleasure afforded by the seat, offers people the opportunity to rest while providing places to socialise [43].

Second, create opportunities for people to socialise around water, through walk-and-talk social groups, wildlife-watching, and boat trips. Such activities foster socialisation but can also contribute to increased physical activity levels. Walking groups have both physiological and psychological health benefits [44]. Research has pointed to the wellbeing benefits of routine encounters with wildlife [45]. Pleasure boating is an enjoyable social activity allowing people to see their surroundings from an alternative perspective. However, boats can have adverse environmental impacts, which must also be considered when considering such activities [46].

Third, foster a sense of community around blue spaces. Supporting local businesses to provide social spaces near water and encourage them to employ local people to strengthen community cohesion. Organising community events such as litter picks, art exhibitions and walking groups can bring people together and create a shared experience and identity. Incorporating public art and installations in or around blue spaces can help create a sense of place and identity and encourage people to engage with the environment in a creative and meaningful way. Long rivers have been found to inhibit social porosity, the degree to which people can be social in a neighbourhood, negatively affecting social cohesion, but bridges may lessen this impact [47].

Providing opportunities for individuals to volunteer and contribute to the upkeep and maintenance of blue spaces can build a sense of ownership and responsibility among community members. Volunteering has been found to improve wellbeing by building self-esteem, self-efficacy and social connectedness [48]. Volunteering in nature can also support peoples' reintegration into society following challenging life events or mental health problems [38].

### 4.3 Stress reduction

Research has consistently shown that being near or interacting with blue spaces can have a positive impact on mental health, reducing stress, anxiety, and depression [10,17]. Other studies have found that exposure to blue space can improve mood and increase feelings of relaxation and calmness [18,49,50]. We propose three policy actions to make blue spaces work for mental health and wellbeing.

First, build infrastructure that supports positive mental health. Engage communities with local wildlife through informative signage and create waterside community gardens for nature connection and collaborative activities [51]. Encourage community ownership of these spaces to foster pride and responsibility. Ensure inclusive, well-lit designs to welcome everyone.

Second, integrate structured therapy spaces, like walk-and-talk or outdoor therapy near blue spaces. In areas that are safe to bathe, water-based therapies can utilise water's calming effects for relaxation and stress reduction. Outdoor talking therapy offers a change of environment while connecting individuals with nature [52].

Third, protect and preserve blue spaces as therapeutic landscapes. Corporate social responsibility volunteers and community payback initiatives can maintain these spaces. Corporate volunteering benefits both companies, individuals and the environment, demonstrating commitment to sustainability and enhancing natural areas. Community payback can contribute to cleaner blue spaces through activities like litter picks and gardening.

### 4.4 Environmental quality

Planetary health and public health are interconnected. We proposed three policy actions to leverage blue spaces to improve the environment, which can positively impact public health and reduce the carbon footprint of our healthcare systems.

First, ensure interventions for human health synergistically work for planetary health. This means reducing the carbon footprint and promoting sustainable practices when designing and integrating new infrastructure into natural environments. Also, offsetting the negative environmental impacts of new infrastructure by incorporating nature-based solutions, including green roofs, rain gardens or permeable pavements, can help achieve synergistic benefits for people and the planet.

Second, ecosystem services play a critical role in preventing the spread of disease and promoting human health. Natural water filtration systems remove pollutants, reducing the risk of waterborne illnesses. Preserving and restoring wetlands enhances these services, and can also improve air quality, reduce flood risk, mitigate urban heat island effects, and enhance the overall liveability of cities.

Third, provide opportunities for environmental conservation and hydrocitizenship, through initiatives like litter pick and rewilding. Encouraging community stewardship fosters responsibility and pride in blue spaces.

## 5 Conclusion

We co-created actions that can be implemented by both those responsible for preserving and revitalising blue spaces and those working in public health. The actions aim to deliver mutual benefits for population and planetary health and are integrated into a whole-system action plan.

We recognise that public health will rarely be the primary driver of regeneration so a focus on systems approaches and maximising co-benefits is particularly important. A systems approach coupled with intensive genuine community engagement, is vital, particularly in areas of deprivation with the greatest levels of vulnerability to poor mental and physical health. The recommendations allow actions to be designed to deliver public health co-benefits regardless of their primary purpose.

While many actions have already been made to enhance blue spaces, often, these are piecemeal and have not engaged the necessary collective of stakeholders in the process. This can lead to actions failing as they do not suit the needs of the local population. Understanding locals' needs and wants is crucial when considering the revitalisation and management of blue spaces. Given the global harm caused by poor mental, social, and physical health, there is a significant opportunity to leverage the benefits of nature to enhance our overall wellbeing.

## Ethics approval

Glasgow Caledonian University School of Health and Life Sciences Ethics Committee approved this research (HLS/PSWAHS/19/208). All participants provided informed consent prior to participating in any co-creation sessions and/or follow-up interviews.

## Funding

This research did not receive any specific grant from funding agencies in the public, commercial, or not-for-profit sectors.

## Conflict of interest statement

The authors declare that there is no conflict of interest associated with this research.
