# Supplementary material for: Leveraging blue spaces for public health: Co-creating a whole-system action plan
Source: Public Health Pract (Oxf). 2025 Oct 4;10:100665. doi: 10.1016/j.puhip.2025.100665 (PMC12550573; doi:10.1016/j.puhip.2025.100665)
Supplement: Multimedia component 1 [file mmc1.docx]

Supplementary Information 1

Narrative Summary of the Systems Map

**Leveraging Blue Spaces for Public Health: Co-creating a whole-system action plan**

**Public Health Journal**

**Niamh Smith ^1 *^, Michail Georgiou ^2^ , Deryck Irving ^3^ and Sebastien Chastin ^1,4^**

^1^ School of Health and Life Sciences, Glasgow Caledonian University, Glasgow G4 0BA, UK

^2^ Urban Big Data Centre, University of Glasgow, Glasgow G12 8QQ, UK

^3^ Hydro Nation Chair Research & Innovation Programme, University of Stirling, Stirling FK9 4LA

^4^ Department of Movement and Sports, Ghent University, Watersportlaan 2, 9000 Ghent, Belgium

* Corresponding Author: Dr Niamh Smith; niamh.smith@gcu.ac.uk; Glasgow Caledonian University, Cowcaddens Rd, Glasgow G4 0BA, United Kingdom

The full system map consists of 137 variables and 220 causal linkages. The variables are represented by boxes of text in the system map. Arrows are used to show the causal relationships between two variables. The variable at the tail of the arrow may cause an effect on the variable at the tip. There are two types of causal relationships: positive and negative. In a positive relationship, both variables change in the same direction. For example, if variable A (at the tail) increases, then variable B (at the point) will also increase (Figure 3). Conversely, if A decreases, then B will also decrease. In a negative relationship, variables change in opposite directions. For example, if B increases, A will decrease, and if B decreases, A will increase (Figure 3). Feedback loops are circular causalities, for example, where the arrows connect variables A to B and then back to A (Figure 3). They are a crucial component of system maps as they aid in understanding the dynamic behaviour of the system.


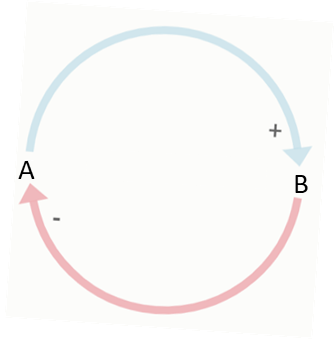


Figure 3 – Positive and negative causal relationships creating a feedback loop

The basic topography of the system map is a core of four variables which we know to be mechanisms through which blue space influences health. All findings reported in this paper are from interviews and co-creation sessions with stakeholders operating around the canals and other waterways in Scotland. Becoming familiar with the map requires breaking it into digestible parts, the mechanisms. Within our map, these four mechanisms are how urban blue spaces can promote physical activity, promote social interaction, contribute to a healthy environment, and help reduce population stress. A periphery of interconnected variables surrounds these core mechanisms. These variables either directly or indirectly contribute to how the blue spaces affect population health, and the whole map reflects the complexity of the relationships between urban blue spaces and health. We discuss each mechanism's critical factors, connections, and loops. Figure 4 shows the full map, coloured by mechanism. An interactive version of the map can be viewed at (removed for anonymity)


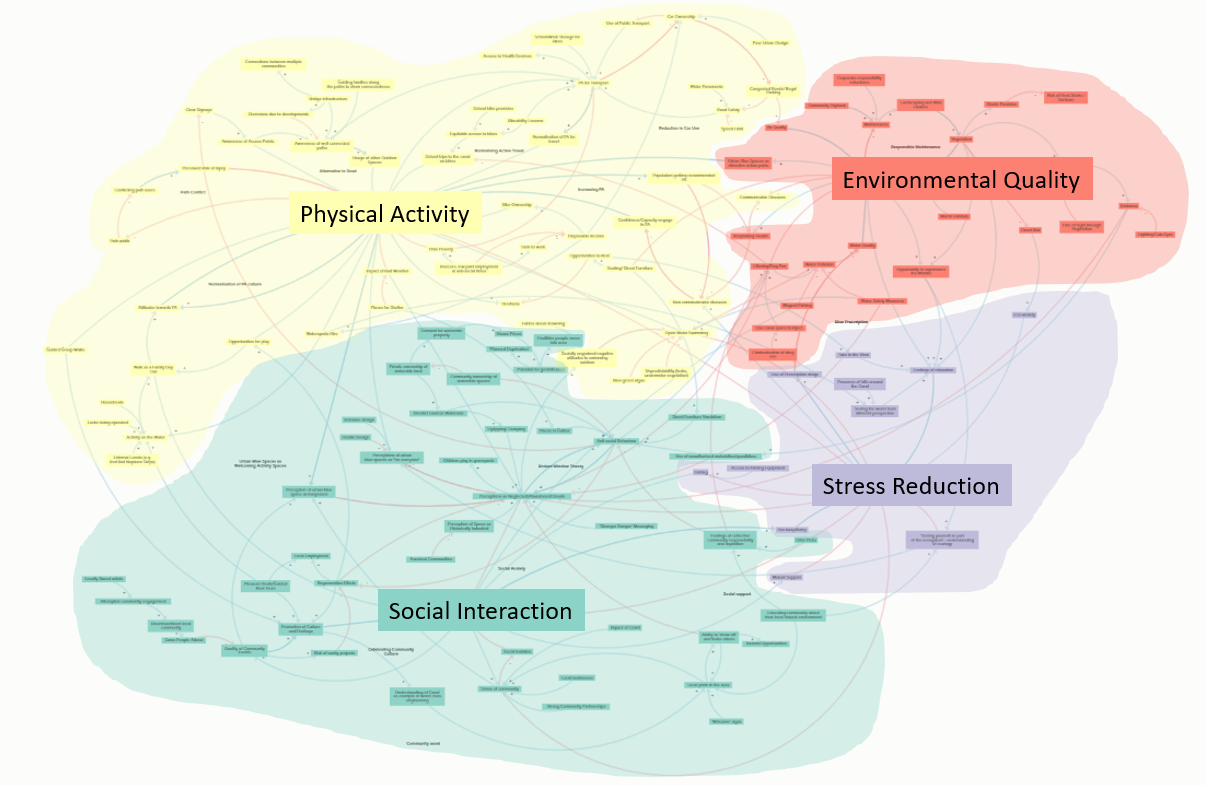


Figure 4 - System map of the factors which contribute to blue space relationship with population health

## Physical Activity

The physical activity cluster contains 55 variables which reflect the enablers and disablers of using the canal for physical activity.

Physical activity is very beneficial for health, and urban blue spaces provide an opportunity to engage in lots of different forms of physical activity, including walking, cycling and running along the towpath and watersports along the water itself.

Many benefits are associated with higher physical activity levels, including reducing respiratory health problems and non-communicable diseases. Non-communicable diseases also decrease people's confidence and capacity to engage in physical activity, leading to them not being as physically active. Respiratory health can also affect infectious diseases, as seen during the COVID pandemic. Similarly, poor environmental quality can also exacerbate communicable diseases. Physical activity also connects to the socialisation section of the map as numerous forms of physical activity can be enjoyed in the company of others, fostering social interactions and connections.

Play is a valuable form of physical activity for children. If there are spaces for children to play, people may see a walk along the canals as a good family day out. This may change attitudes towards physical activity as people perceive it to be more social and family engagement rather than a strict form of exercise, leading to more families walking, so we may see a normalisation of the physical activity culture loop emerge. Guided group walks regularly use the canals, allowing people to do low-intensity physical activity. Some of the guided walks are themed and promote the culture and heritage of the canals, so as well as being physically active, people are learning about their area, which can have other benefits, described later.

Having space to shelter around the canals may affect how people use them. For example, there are high levels of rainfall in Glasgow, and the canals have very few built shelters to allow for rain shelter. More spaces for people to take shelter from the rain may benefit groups of cyclists, walkers and wheelers. However, it is also noted that increased street furniture may give rise to higher levels of vandalism and antisocial behaviour, as they could become areas for people to crowd and drink.

Path width was cited as something that can negatively impact physical activity levels. When the path width is narrow, conflict can increase across different path users (e.g. Cyclists and dog walkers), increasing the perceived risk of injury and leading to some people choosing not to partake in physical activity. Other infrastructure around the canal can affect physical activity levels, including clear signage that increases awareness of where the access points are and of the interconnectedness of the canal paths, for example, to other local waterways and green spaces. These interconnected paths provide a network of routes for pedestrians and cyclists as an alternative to the road.

The newly constructed Stockingfield Bridge increases the paths' connectedness and allows more communities to readily access the canal path and partake in physical activity. However, this development was potentially confusing for locals; the diversions during the bridge construction made it challenging to identify access points. Nevertheless, it is hoped that the bridge will increase the connections between multiple communities, improving social interaction and physical activity.

Cycling is popular on the canals, and cycling for recreation can be a form of active travel. A 'normalising active travel' loop emerged where if more people are physically active along the canal and use physical activity for transport, this may prompt others also to be more active.

Equitable access to bikes may promote physical activity among children. Some schools offer bike-share schemes and organise cycle safety sessions, like Bikeability, which boosts skills and confidence. Such resources and upskilling allow schools to take children on bike trips along the canals. However, this is hampered by littered needles that have been used to inject drugs. A much broader topic discussed as part of this project was the criminalisation of drug use and the lack of safe drug consumption facilities that lead to vulnerable individuals using the spaces surrounding the canals to inject substances like heroin. People also use the canals for drinking alcohol, and so sometimes, there can be smashed glass littering the paths. Dirty needles and glass on the canal pose a severe danger to the public, directly causing injury. Also, people may be indirectly harmed as the sharp objects can puncture tyres on bicycles and prams, thwarting physical activity.

The canals were seen as a valuable route for people to actively travel to access health services like doctor surgeries and pharmacies. It was reported in our co-creation workshops that people travelled along the canals to access these services.

Having opportunities for people to store their bikes at school and work allows people to be more likely to use their bikes for active travel to work leading to greater physical activity levels.

If people use bikes more, there may be less need to own a car. High levels of car ownership can lead to increased congestion and illegal parking and loading, which negatively affect air quality, leading to poorer overall environmental quality. In addition, congested streets also pose a road safety risk for non-drivers. Illegal parking around schools was a genuine safety concern for why some children, young people and parents do not travel to school on foot; they feel unsafe.

Glasgow's urban planning has led to the city being intersected by motorways and expressways that divide communities and discourage active travel and public transport (Kintrea, 2019). The regional focus on road infrastructure, and the minimal investment in transport within the city over several decades, means that it is often easier and quicker to commute from affluent middle-class suburbs to the city centre than it is from places in the inner city (Kintrea, 2019). One participant described this as an issue of social justice.

Efficient public transport encourages people to use it more, in conjunction with cycling and walking where appropriate. Other factors that play into physical activity for transport include road safety. It is unlikely that people can solely use the canals as a route for active travel; people need interconnected, active infrastructure. Wider pavements, segregated cycle lanes and speed limits for vehicles may contribute to increased perceptions of the safety of urban streets for non-car users.

Other factors that contribute to physical activity levels include the time people have to participate in that physical activity. In North Glasgow, insecure and low-paid employment at antisocial times can mean that people are less likely to participate in physical activity. It was reported in our research that some people have to take taxis to work because of the time that they work, it would be too dark to travel along the canals or walk, and buses do not frequently run through the night. Such workers have lower disposable income to partake in many types of physical activity. Bikes are expensive and may not be a priority for many people. Similarly, such demographics may not afford to own a car. This exposes issues of equity in access to the resources needed for active travel.

The physical environment affects physical activity in several other ways, such as the amount of seating and street furniture present along the canal. It was reported in our research that without opportunities to rest, some people might feel too tired and, therefore, not be as likely to be to partake in physical activity.

As well as walking and cycling, some participants discussed open-water swimming as a form of physical activity that could take place on the canals. Open-water swimming has been found to reduce stress and be an excellent form of physical activity. However, several factors deterred swimmers, including the unpredictability of the locks, for example. Even when no boats are around, locks can slam shut with force created by water pressure. Blue-green algae can also be present on canals, which is dangerous to both humans and animals, causing skin and stomach problems and more severe illnesses. Additionally, the murkiness of canals means that swimmers cannot see what is beneath them and, therefore, may get tangled in undergrowth. Our participants also referenced societal factors that can influence behaviours, including socially ingrained negative attitudes to swimming outdoors and stories passed down through generations of the dangers of drowning in the canals.

Similarly, water pollution can play a role in water quality. Polluted water may dissuade swimmers. If there are designated safe, clean swimming areas, more people may choose to.

As well as swimming, people may participate in other types of water sports like kayaking and canoeing, contributing to increased physical activity. However, it is noted here that water sports can be an expensive hobby, requiring higher disposable income levels. People commented on enjoying seeing activity on the water, like kayaking and canoeing, but also enjoyed viewing the locks being operated and external events like the Red Bull Neptune Steps race, a race that takes place in the Maryhill Locks in Glasgow, Scotland, where participants must swim through a series of 7 lock gates, climb over obstacles, and finish with a final swim to the end. The increased activities all contribute to the canal appearing as a desirable destination for days out, which, in turn, may attract people to the area to start engaging in lower-intensity physical activities, like walking.

## Social Interaction

Another mechanism through which blue spaces can affect health is that they allow people to interact socially with one another as they are within those spaces. The Social Interaction cluster contains 46 variables which reflect how the relationship between blue space and health is influenced by how people interact with one another. Social interaction is strongly connected to health and wellbeing (Umberson and Montez, 2010), and the converse is that social isolation can be damaging to health, particularly for high-risk individuals, including older adults (Lee *et al.*, 2021). This has been apparent during the COVID-19 pandemic lockdowns (Razai *et al.*, 2020).

Increased social interaction along the canals may foster a sense of community. Other things that promote a sense of community within the local area include local businesses that operate around the canals and strong community partnerships of people working around the canal network. Local pride in the area can also foster a sense of community. Features like welcome signs on the canal or 'Welcome to Cadder' signage can foster local pride in the area and contribute to increased community cohesion (Figure 5).


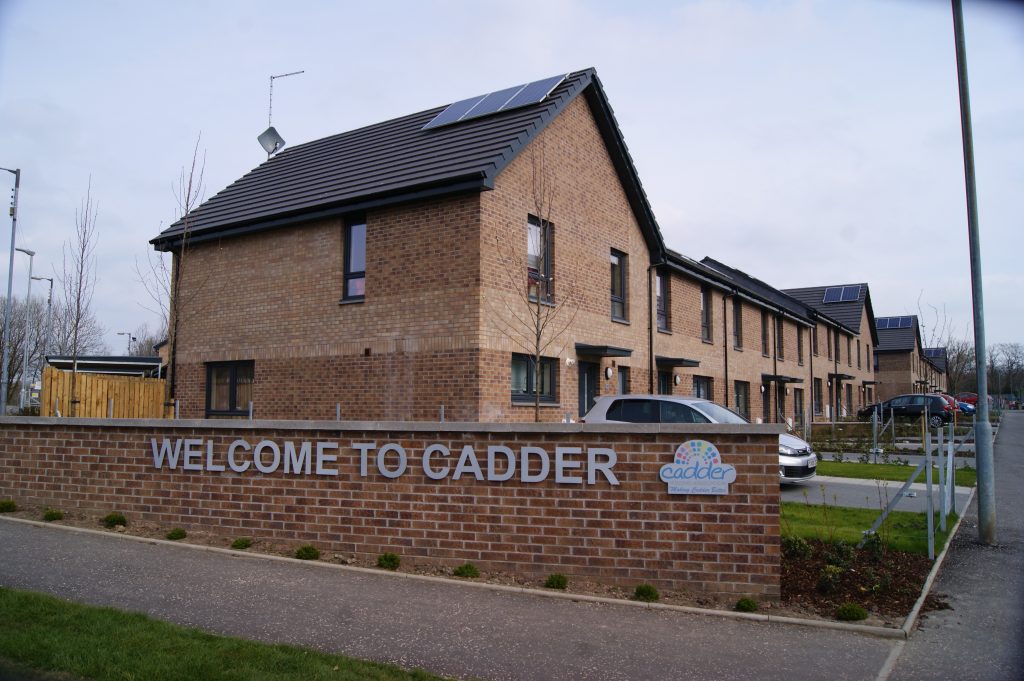


Figure 5 - 'Welcome to Cadder' signage to increase local pride and make the area inviting.

The transient nature of the communities around the canal affects how different people perceive this space. There are large transient communities, with the North of Glasgow being home to many Eastern European migrants (Kay and Trevena, 2019). Interestingly, such community members do not have the same ingrained negative attitudes towards the canal as born and raised locals, leading them to use the space more (Kay and Trevena, 2019).

People reported local pride in having the confidence to show off and invite others to come and see where they live, especially around the recently regenerated Hamiltonhill Claypits Local Nature Reserve (The Claypits) (Figure 6). The Claypits provided an attraction where people felt proud to invite their friends and family to the local area who otherwise would never come to the north of Glasgow. This ability to show off the canals as a destination also increases the likelihood of tourism opportunities. Things that can foster local pride in an area include encouraging local community members to engage in the local natural environment, allowing them to see themselves as an intrinsic part of the ecosystem, and understanding how they 'fit' within the ecology around them.

Figure 6 - Recently regenerated Hamiltonhill Claypits Local Nature Reserve


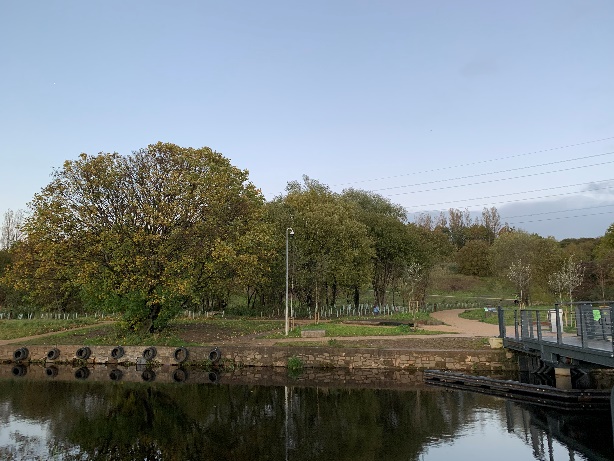

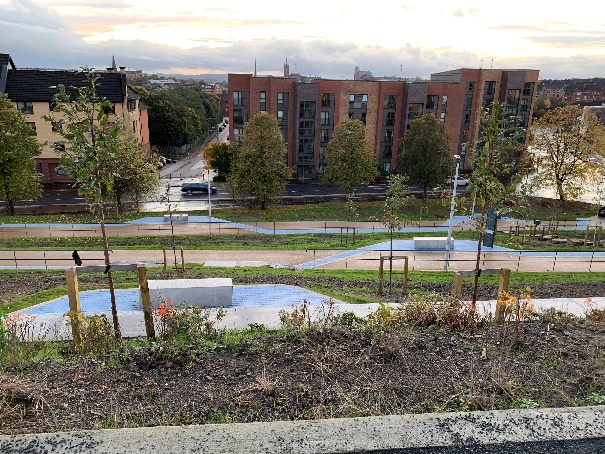


Seeing yourself as part of the ecosystem may make the canal space feel like a space for everyone. Learning about the local natural environment is also really important. It increases feelings of collective community responsibility and aspiration, potentially reducing antisocial behaviour; people feel responsible, ownership, and pride in their local area and therefore want to preserve and look after it. Conversely, feeling that a space is 'not for you' and that you are not welcome can lead to antisocial behaviour. Increased community ownership of canal-side spaces may make antisocial behaviour less likely. Things that contribute to antisocial behaviour include behaviours such as using unauthorised motorbikes and quad bikes, drinking alcohol and leaving empty bottles and cans, injecting drugs and littering the paraphernalia, and vandalism.

The development of the canal side carries the risk of gentrification and the potential for private ownership of waterside land. This could result in an increased demand for waterside properties, leading to rising house prices. The consequences of such changes can be detrimental, causing the displacement of current residents and attracting a wealthier and healthier population to the area, without necessarily improving the overall health of the existing community.

Furthermore, privately owned waterside land poses another concern. If left abandoned or neglected, it can become derelict, leading to a rise in fly-tipping and dumping activities. This neglect further reinforces the perception that the canals are disregarded, abandoned, and unsafe. Negative perceptions of the canals were a recurring theme among the participants. In particular, a teacher pointed out that children do not feel safe along the canals and prefer to play in local graveyards due to better lighting and a greater sense of security compared to the canal areas. The perception of canals as unsafe is also perpetuated by the "Stranger Danger" messaging taught in schools and passed down through generations.

If parts of the canal are left neglected, it could lead to more vandalism and antisocial behaviour. 'Broken window' theory explains that when there are signs of antisocial behaviour in a neighbourhood, it can create an environment that encourages more disorder (Barishansky, 2010). So, if certain areas of the canal look rundown, abandoned, and unsafe, it's more likely that there will be additional antisocial behaviour. This can create a cycle of negative behaviour and deterioration of the space.

The idea that the canal space is for everyone came up in co-creation workshops. However, some people identified the idea of hostile design, where infrastructure is built to deliberately exclude specific demographics of the population. Such design can lead to people feeling unwelcome along the canals. Some people have commented that although the area around a watersports centre is a public space, its design can make it feel unwelcoming to the general public, who may feel excluded unless they can afford to participate in the watersports activities offered there. Inclusive design, therefore, is essential as that boosts this perception that the canal is a space for everyone. Conversely, when spaces are busy and well-used by people' like them', people feel safer. This is another example of a reinforcing loop whereby the more people on the canal, the more likely others will choose to join.

The idea of promoting the canals, culture and heritage was seen as really important. Pleasure boats and guided boat tours along the canal lead to increased activity on the water which can lead to many other knock-on effects. But they can also lead to local employment. Similarly, if done well, regeneration efforts around the canals can promote culture and heritage and increase play opportunities. However, it was also noted that people did not want to see vanity projects along the canals. The quality of community engagement and the quality of community events around the canal were critical to mitigating this. Factors around the quality of these events included people's ability to engage. For example, locally-based artists may try to engage community members. However, that may lead to disenfranchised local community members as the artists may not have the skills to engage authentically and build trust. That means that perhaps the same people attend the consultation groups, which leads to a lower quality of this consultation and an increased risk of vanity projects prevailing. On the other hand, many community artists can have the expertise and local knowledge to engage effectively with communities. Finally, promoting the culture and heritage of the canals is significant, and increasing knowledge that the canals are an example of world-class engineering can foster increased pride in the local area.

## Stress Reduction

The Stress Reduction Interaction cluster comprises 12 variables that emphasise the impact of blue spaces on health, in relation to stress reduction and mental wellbeing. Time spent along the canals and other blue spaces can lower stress levels, owing to the therapeutic properties of water environments. Engaging in activities such as fishing along the canals not only promotes social interaction but also helps alleviate stress.

The concept of a "Blue prescription" is gaining traction as healthcare professionals recognise the therapeutic benefits of spending time in nature, particularly in blue spaces like oceans, rivers, and lakes, for enhancing mental health and overall wellbeing. This approach is being increasingly considered as a potential intervention for mental health conditions in some patients. By encouraging people to engage with nature and blue spaces to prevent and treat mental health conditions, there is the potential to reduce reliance on prescribed medication. At a population level, this could lead to a decrease in the presence of prescription drugs entering watercourses over time, ultimately contributing to the improvement of water quality. Increased blue prescription may also decrease the carbon footprint associated with pharmaceutical production, transportation, and disposal, contributing to decarbonising healthcare systems. This feedback loop connects the stress reduction and environmental quality sections of the systems map, highlighting the interconnectedness of these issues.

Social interaction and stress reduction are interconnected. Increased social interaction can lead to the concept of mutual social support, which can lead to reduced stress and, in turn, people who are less stressed are more likely to want to and have the capacity to interact with people socially, creating a reinforcing feedback loop.

However, if the canal is too busy and many people compete for space, some individuals may experience increased stress. Similarly, the perceptions of the canals as being neglected, abandoned and unsafe are likely to heighten people's stress.

Opportunities that allow for relaxation are likely to make people feel less stressed. Many things can influence these feelings of relaxation; for example, when people can sit along the canal, they can take time to become grounded in their surroundings, take in the view and have increased feelings of relaxation. Some participants noted the hills around the canal, like the viewpoint at the Claypits, which allows you to see Glasgow from a different perspective, reflect and recharge, perhaps increasing relaxation and reducing stress.

It was also noted that water sports, like kayaking and canoeing, allow people to see the world from a different perspective, which can also lead to feelings of relaxation.

## Environmental Quality

The Environmental Quality cluster contains 24 variables highlighting the interplay between the environment and health.

The quality of the natural and built environment can affect how relaxed people feel. High environmental quality with ample vegetation contributes to safe and healthy habitats for wildlife, which people enjoy interacting with and encourage people's connectedness to the natural world. The trees and bushes lining the canal sequester carbon dioxide and contribute to cleaner air while also being visually appealing. For example, the vegetation in the marshlands at the Claypits sequester carbon dioxide, while the anaerobic soils store carbon, contributing to improved environmental quality. Poor air quality harms people even if they do not have lung disease, but it is hazardous for people with asthma, COPD, and other respiratory ailments. Cleaner air, therefore, benefits everyone and may reduce the health implications of respiratory diseases. The creation of an attractive, usable corridor along the canal offers routes which are away from busy and polluted streets.

Increased vegetation along the canals can effectively intercept and store runoff before it reaches the canal channel, reducing the likelihood of flooding. Risks of flooding may contribute to people's stress levels, and so measures to tackle urban response to climate change may lessen the burden of eco-anxiety and generally reduce stress. Vegetation also provides shade, affecting our health by reducing the risk of heatstroke and sunburn. Additionally, nature-based solutions like the Smart Canal in North Glasgow can integrate water management and the canals water levels to reduce risk of flooding.

However, vegetation can also create a 'dark screen', contributing to the dark canal. Maintaining the vegetation surrounding the canals in such a way as to provide a line of sight through it may lead to it feeling brighter, less enclosed and safer.

The canal is rich in trees and plant life, which needs careful management to allow everyone to enjoy them. A few different options for types of maintenance were discussed, including organised programmes incorporating painting, litter picks, vegetation removal and gardening for people serving Community payback orders in the Criminal Justice system. Additionally, some corporate social responsibility volunteers from local businesses have been involved in maintenance projects around the canals, helping with landscaping and wildflowers and improving the canal's aesthetics.

Lighting and cats eyes along the canals may be beneficial to reduce the darkness and increase usage. Some sections of the canal towpath have ground level lighting, yet some areas remain very dark.

Litter was discussed as contributing to reduced physical activity levels above. In addition to glass and needles, plastic litter can be dangerous and unsightly for both people and animals. Litter from nearby fast-food chains was referenced, and people also noted the total absence of bins along the paths. Additionally, dog faeces carries diseases that can be transmitted to humans and other animals; Salmonella, E. Coli, and Campylobacter are bacteria even excreted in that of healthy dogs. Dog faeces is also toxic to the environment, and water supply contamination can lead to an overgrowth of algae, killing wildlife.

Lastly, by shifting the narrative surrounding canals and presenting them as public urban parks rather than mere waterways running through the city, we can reframe their perception as attractive natural destinations within the bustling, densely developed urban environment.
